# Quincy Medical Society

**Published:** 1871-07

**Authors:** Addison Niles

**Affiliations:** Secretary


### QUINCY MEDICAL SOCIETY.

The Annual Meeting of the Quincy Medical Society was held at the office of Dr. A. Niles, in the city of Quincy, May 9th, at 10 o'clock A.M.

The following members were present, *viz.:* Drs. J. M. Grimes, J. T. Kinsler, A. Niles, L. Tibbets, C. A. W. Zimmermann, Sen., and William Zimmermann.

The meeting being duly organized, Dr. J. H. Reynolds, chairman, the following officers were elected for the ensuing year:

*President*—Dr. C. A. W. Zimmermann, Sen.

*Vice-President*—Dr. A. J. Miller.

*Secretary*—Dr. A. Niles.

*Censors*—Drs. L. H. Baker, P. A. Marks, L. Tibbets, Wm. Zimmermann, and J. M. Grimes.

On motion, Dr. C. A. W. Zimmermann, Sen., Samuel Tibbets and A. Niles were elected delegates to attend the next meeting of the State Medical Society.

Dr. J. M. Grimes was appointed orator for the next meeting.

Drs. Wm. Zimmermann, L. H. Baker, and L. Tibbets were appointed by the President a committee to report at the next annual meeting upon the diseases prevailing in Adams County the present year.

At two o'clock P.M. the Society convened, pursuant to adjournment, for the purpose of hearing and determining the charges which had been preferred against Dr. J. F. McCormick. The doctor not appearing, the Secretary, after waiting a reasonable time for him, proceeded to read the following bill of charges, which had been preferred against him in due form:

" *Quincy, Ill., April 13th, 1871.*

" Dr. J. F. McCormick,

" Dear Sir,—We, the subscribers, members of the Quincy Medical Society, moved solely by a desire to sustain the respectability and honor of the profession, accuse you of disregarding the code of ethics and bye-laws of the said Society, of which you are a member, by the publication of a pamphlet intended for popular reading, bearing date Quincy, Ill., 1871, entitled 'A New Treatise on the Cause and Cure of Pulmonary Consumption and Kindred Diseases,' and likewise by the advertisements within said work and on its cover, and in the daily prints of this city.

"We accuse you, first, of violating the provisions of the code of ethics of the American Medical Association contained in art. 1, sec. 1 and 3, entitled 'The duties of physicians to each other and to the profession at large.'

"Second, with directly transgressing the bye-laws of the Quincy Medical Society, by the publication of advertisements in the daily prints of this city, as well as in said pamphlet. See p. 11, art. 17 of the bye-laws.

"You, Dr. J. F. McCormick, are hereby notified to appear at the next annual meeting of the Quincy Medical Society and answer to the above accusations, which will then be preferred against you, said meeting to be held at the office of Dr. A. Niles, on the 9th day of May next, at 2 o'clock P.M.

"SAMUEL A. AMERY. ADDISON NILES.
"C. A. W. ZIMMERMANN, SEN. P. A. MARKS.
"J. T. KINSLER. WM. ZIMMERMAN."

"I do hereby certify that the above is a true copy of the charges presented against Dr. J. F. McCormick, and that a copy of said charges was personally given to him by myself, as the bye-laws direct, on the 13th day of April, 1871.

"ADDISON NILES, Secretary."

The accused failing to appear or to present to the Society any defence, or render any excuse for the neglect, the members present having examined the evidence adduced to sustain the charges proceeded to vote upon the question: Are the charges sustained by the evidence? The vote being taken every member present voted in the affirmative, excepting Dr. J. H. Reynolds, who declined to vote on account of unsettled business relations with the accused.

Dr. C. A. W. Zimmermann, Sen., then moved that Dr. J. F. McCormick be expelled from the Quincy Medical Society. The motion being seconded, was put by the President, and carried by an unanimous vote.

On motion, resolved that the Secretary be directed to notify the State Medical Society of the expulsion of Dr. J. F. McCormick, and likewise furnish the CHICAGO MEDICAL EXAMINER with an abstract of the proceedings of this meeting for publication.

ADDISON NILES, Secretary.

